# Factors Influencing Liver Abnormalities in Psoriatic Arthritis Patients: A Comprehensive Study

**DOI:** 10.31138/mjr.050723.fla

**Published:** 2023-08-27

**Authors:** Ping Seung Ong, Lay Kim Tan, Hasnah Mat, Najjah Tohar, Abdul Muhaimin Fathi, Nia Maslia Atiera Kosenin, Muhammad Najmi Budiman Naim, Rafiqah Farhanah Redzuan, Nur Iffah Ab Rani, Najiha Arrissa Norhisham, Wahinuddin Sulaiman

**Affiliations:** 1 Rheumatology Unit, Department of Medicine, Hospital Raja Permaisuri Bainun, Jalan Raja Ashman Shah, Ipoh, Perak, Malaysia,; 2 Sector for Biostatistics & Data Repository, Office of NIH Manager, National Institutes of Health, Ministry of Health Malaysia, Selangor, Malaysia,; 3 Faculty of Medicine, University Kuala Lumpur Royal College of Medicine Perak, Perak, Malaysia

**Keywords:** disease duration, diabetes mellitus, liver enzymes abnormalities, non-alcoholic fatty liver disease, psoriatic arthritis

## Abstract

**Objective:**

The aim of this study was to establish the incidence of liver abnormalities in psoriatic arthritis patients and identify the factors that contributed to this condition.

**Methods:**

This is a longitudinal cohort study. Psoriatic arthritis (PsA) patients with liver enzymes abnormalities were identified. Our control group consisted of PsA patient from the same cohort who had no history of liver abnormalities. Factors associated with liver abnormalities were identified using univariate and multivariate analysis.

**Results.:**

A total of 247 of PsA patients were included and out of those, 99 developed liver enzymes abnormalities. The mean age of the patients was 56 years old (±13.5) with 56.1% female and 39.4% Indian descendants. The univariate logistic regression demonstrated that disease duration of PsA (OR=1.06, 95% CI=1.01 - 1.10, p=0.012), diabetes mellitus (OR=2.16, 95% CI=1.26 - 3.70, 0.005) and non-alcoholic fatty liver disease (NAFLD) (OR=3.90, 95% CI = 1.44 - 10.53, p=0.007) were associated with abnormal liver function in PsA patients. No association was found with both conventional synthetic disease-modifying antirheumatic drugs or biologics.

**Conclusion:**

Liver enzymes abnormalities in PsA patients were linked to disease duration, diabetes mellitus and NAFLD. For these high-risk populations, vigilant monitoring of liver function tests is vital for early detection and intervention.

## INTRODUCTION

Psoriatic arthritis (PsA) is a type of arthritis associated with psoriasis that affects both spinal and peripheral joints, as well as entheses and dactylitis. Recent research has found a connection between psoriasis and various comorbidities, including metabolic syndrome and cardiovascular disease.^1^ Studies have shown that non-alcoholic fatty liver disease (NAFLD) is the most prevalent liver abnormality in individuals with psoriatic disease with both obesity and metabolic syndrome as risk factors.^2,3^ NAFLD ranges from simple steatosis to steatohepatitis, cirrhosis, and hepatocellular carcinoma, in the absence of excessive alcohol intake.^4^

It is important to closely monitor patients with PsA who are being treated with non-biological and biological disease-modifying antirheumatic drugs (DMARDs) due to concerns about their safety including liver enzymes elevations. Although these elevations are mostly asymptomatic, persistent elevations have been shown to correlate with histopathological changes of fibrosis assessed by liver biopsy. ^5^ In addition, factors such as non-steroidal anti-inflammatory drugs (NSAIDs), obesity and alcohol consumption may also contribute to liver abnormalities.^6,7^ Our objectives were to determine the incidence of liver abnormalities and factors contributed to this condition among PsA patients.

## MATERIALS AND METHOD

This is a longitudinal cohort study of PsA patients followed up by Rheumatology Division, Hospital Raja Permaisuri Bainun Malaysia from 2015 until 2022. Eligible patients included those fulfilled the ClASsification for Psoriatic ARthritis (CASPAR) criteria for PsA.

The medical history of patients in the study included questions about the PsA duration, ethnicity, working status, education level, smoking history, alcohol consumption, presence of comorbidity, concomitant drugs for PsA and other illnesses. During the clinical examination, the patients’ height, weight, and blood pressure were measured. The body mass index (BMI) was calculated using the patient’s weight and height, using the following formula: BMI = weight in kilograms divided by height in meters squared. A BMI of 30 or higher is considered obese.

Liver test abnormalities was defined by aspartate transaminase (AST) and/or alanine Transaminase (ALT) above the upper limit of normal (ULN) which is ≥ 50 IU/L. Once patients with new liver function test abnormalities were identified, we tracked these patients at their next 2 follow up to assess for persistent abnormalities. In our study, we implemented assessment for PsA patients at intervals ranging from 1 to 6 months bases on the condition of the patients. These assessments involved comprehensive physical examinations and laboratory evaluations tailored to each patient’s condition.

Liver diseases collected included NAFLD and drug -induced liver injury, The diagnosis of NAFLD was confirmed by imaging study such as liver ultrasound or computed tomography scan . Drug-induced liver injury in this study was made based on intake of a potential drug at least 1 visit prior to detection of liver abnormality, exclusion of other potential causes of liver damage and resolution of liver abnormalities after discontinuation of the potential drug.

### Statistical analysis

Descriptive statistics were employed to depict the sociodemographic characteristics, lifestyle risk factors, comorbidities, clinical presentation, and treatment regimens. Using logistic regression analysis, we investigated how sociodemographic factors, clinical factors, manifestations, and treatment regimens were related to the risk of liver abnormalities among patients with PsA. Factors with p-value of equal or less than 0.25 in univariate logistic regression analysis were selected for multivariate logistic regression analysis. The generalised estimating equation (GEE) was employed to produce regression estimate when analysing the repeated measures of the AST and ALT levels in the PsA patients treated with combination of methotrexate (MTX) and leflunomide (LEF) at three different visits. Age, gender, disease duration, smoking status, obesity and alcohol consumption status were included as covariates in the GEE analysis. The Beta coefficient (B), with the respective 95% confidence intervals (95% CIs), were calculated. A p-value of less than 0.05 was considered significant. All statistical analyses were conducted using the IBM Statistical Package of Social Sciences (SPSS) for Windows version 28.0 (IBM Corp., Armonk, NY, USA).

## RESULTS

A total of 247 psoriatic arthritis patients visited the rheumatology clinic at the Raja Permaisuri Bainun Hospital, Ipoh, Perak, Malaysia from 2015 until 2022. Ninety-nine PsA patients had abnormal liver enzymes while the remaining 148 PsA had normal liver enzymes. The mean age (±SD) of the patients was 56 years old (±13.5). When stratified by liver enzymes abnormalities, the mean age of the patients was comparable (abnormal liver enzymes: 56.5 years old (±12.4) and normal liver enzymes: 55.7 (±14.2)) as shown in **Table 1**. Among the 247 patients with psoriatic arthritis, 56.1% were female, while 39.4% were of Indian descent. Upon further stratification by liver enzymes, our data revealed a significant difference in the duration of PsA between patients with transaminitis and those with normal liver enzymes. Specifically, the patients with abnormal liver enzymes had a mean duration of 10.9 years (±7.9), whereas the normal liver enzymes group had a mean duration of 8.6 years (±5.7) (p=0.008) (**Table 1**). Furthermore, we observed a significantly higher proportion of diabetes mellitus (44.4%, p=0.005) and non-alcoholic fatty liver disease (NAFLD) (14.1%, p=0.004) among the PsA patients with transaminitis.

**Table 1.** The characteristics of the psoriatic arthritis patients (n=247), stratified by liver abnormality status.

	**Abnormal liver function (n= 99)**	**Normal liver function (n= 148)**	**p**
**Sociodemographic factors**			
Age, mean (±SD)	56.5 (±12.4)	55.7 (±14.2)	0.635
Sex, n (%)			
Female	51 (51.5)	87 (59.2)	0.235
Male	48 (48.5)	60 (40.8)	
Ethnicity, n (%)			
Malay	32 (33.7)	51 (36.2)	0.405
Chinese	19 (20.0)	39 (27.7)	
Indian	43 (45.3)	50 (35.5)	
Others	1 (1.1)	1 (0.7)	
Education level			
Primary	0 (0.0)	1 (2.0)	0.673
Secondary	5 (19.2)	8 (16.3)	
Tertiary	1 (3.8)	5 (10.2)	
Unknown	20 (76.9)	35 (71.4)	
**Lifestyle factors**			
Ever-smoker, n (%)	60 (60.6)	84 (56.8)	0.548
Obese*, n (%)	22 (29.7)	30 (30.6)	0.901
Ever-drinker (alcohol consumption), n (%)	48 (48.5)	72 (48.6)	0.980
**Clinical characteristics**			
Age at diagnosis, mean (±SD)	45.6 (±12.2)	47.1 (±13.5)	0.397
Duration of PsA, mean (±SD)	10.9 (±7.9)	8.6 (±5.7)	**0.008**
**Pattern of joint involvement**			
Axial	8 (8.1)	12 (8.1)	0.994
Peripheral	92 (92.9)	137 (92.6)	0.915
Peripheral and axial	14 (14.1)	22 (14.9)	0.875
ESR, mm/h, mean (±SD)	42.2 (±31.1)	43.6 (±32.4)	0.740
**Comorbidities, n (%)**			
Diabetes mellitus	44 (44.4)	40 (27.0)	**0.005**
Hypertension	56 (56.6)	73 (49.3)	0.264
Ischaemic heart disease	11 (11.1)	13 (8.8)	0.545
Hyperlipidaemia	49 (49.5)	60 (40.5)	0.165
Cancer	3 (3.0)	3 (2.0)	0.616
NAFLD	14 (14.1)	6 (4.1)	**0.004**
Osteoarthritis	6 (6.1)	15 (10.1)	0.261
Chronic liver disease	12 (12.1)	21 (14.2)	0.640
**csDMARDs**			
MTX	42 (42.4)	117 (57.6)	0.116
SSZ	37 (37.2)	59 (62.8)	0.474
LEF	48 (48.8)	21 (51.2)	0.213
MTX+ LEF	43 (43.0)	118 (57.0)	0.034
LEF+ SSZ	39 (39.5)	69 (60.5)	0.857
LEF+ SSZ+ MTX	41 (41.3)	132 (58.7)	0.199
**Steroid**	11 (44.0)	14 (56.0)	0.673
**NSAIDs**	79 (40.5)	116 (59.5)	0.789
**Biologics#**	18 (47.4)	20 (52.6)	0.319

* Only individuals with obesity data were included in the analysis.

# Include tumour necrosis factor, Interleukin -17 A inhibitor and Interleukin 23p19 inhibitor

ESR: erythrocyte sedimentation rate; PsA: psoriatic arthritis; NAFLD: non-alcoholic fatty liver disease; MTX: methotrexate; LEF: leflunomide; SSZ: sulphasalazine; csDMARDs: conventional synthetic disease-modifying antirheumatic drugs; NSAIDs: Non-steroidal anti-inflammatory drugs.

The results of the univariate logistic regression analysis indicated that the disease duration of PsA (odds ratio [OR]=1.06, 95% confidence interval [CI]=1.01–1.10, p=0.012), diabetes mellitus (OR=2.16, 95% CI=1.26–3.70, p=0.005), and NAFLD (OR=3.90, 95% CI=1.44–10.53, p=0.007) were factors significantly associated with an increased risk of transaminitis among the PsA patients (**Table 2**). In the further multivariate logistic regression analysis, both diabetes mellitus (OR=2.01, 95% CI=1.10–3.68, p=0.024) and NAFLD (OR=5.44, 95% CI=1.79–16.50, p=0.003) were identified as independent risk factors for transaminitis among the PsA patients, as shown in **Table 2**.

**Table 2.** Univariate and multivariate analyses for liver abnormalities among the patients with PsA.

**Variables**	**Crude OR (95% CI)**	**p**	**Adjusted OR (95% CI)**	**p**
**Sociodemographic factors**				
Sex (Male as reference)	0.73 (0.44, 1.22)	0.235	0.70 (0.4, 1.25)	0.230
Ethnicity				
Malay	0.73 (0.40, 1.33)	0.304	1.04 (0.53, 2.05)	0.902
Chinese	0.57 (0.29, 1.12)	0.103	0.72 (0.34, 1.54)	0.399
Indian (R)				
Others	1.16 (0.07, 19.15)	0.916	2.12 (0.08, 53.69)	0.649
Alcohol intake	0.99 (0.60, 1.65)	0.980		
Obesity	0.96 (0.50, 1.85)	0.901		
Smoking	1.17 (0.70, 1.97)	0.548		
Duration of psoriasis, years	1.01 (0.98, 1.04)	0.430		
Duration of PsA, years	**1.06 (1.01, 1.10)**	**0.012**	1.05 (1.00, 1.11)	0.050
ESR	1.00 (0.99, 1.01)	0.739		
Diabetes mellitus	**2.16 (1.26, 3.70)**	**0.005**	**2.01 (1.10, 3.68)**	**0.024**
Hypertension	1.34 (0.80, 2.23)	0.265		
Ischaemic Heart Disease	1.30 (0.56, 3.03)	0.546		
Hyperlipidaemia	1.44 (0.86, 2.40)	0.166	1.10 (0.61, 1.98)	0.763
NAFLD	**3.90 (1.44, 10.53)**	**0.007**	**5.44 (1.79, 16.50)**	**0.003**
Osteoarthritis	0.57 (0.21, 1.53)	0.265		
**Monotherapy**				
MTX	1.75 (0.87, 3.55)	0.119	1.55 (0.57, 4.24)	0.393
LEF	1.53 (0.78, 3.00)	0.215	1.27 (0.58, 2.79)	0.548
SSZ	0.83 (0.49, 1.40)	0.474		
**Combination 2 csDMARDs**				
MTX+ LEF	1.33 (0.66, 2.68)	0.431		
MTX +SSZ	1.03 (0.59, 1.79)	0.923		
NSAIDs	1.09 (0.58, 2.04)	0.789		
Steroids	1.20 (0.52, 2.76)	0.673		
Biologics*	1.42 (0.71, 2.85)	0.321		
Biologics + csDMARDs	1.91 (0.59, 6.17)	0.281		

* Include tumour necrosis factor, Interleukin -17 A inhibitor and Interleukin 23p19 inhibitor.

ESR: erythrocyte rate; PsA: psoriatic arthritis; NAFLD: non-alcoholic fatty liver disease; MTX: methotrexate; LEF: leflunomide; SSZ: sulphasalazine; csDMARDs: conventional synthetic disease-modifying antirheumatic drugs; NSAIDs: Non-steroidal anti-inflammatory drugs.

We conducted further investigation into the influence of conventional synthetic disease-modifying antirheumatic drugs (csDMARDs), specifically the combination of MTX and LEF on the level of liver transaminases in 103 PsA patients. In our observation, there was a trend of higher AST levels at the second (β=1.92, 95% CI=−1.42 – 5.27) and third (β=0.67, 95% CI=−3.21 – 4.55) visits in PsA patients treated with a combination of MTX and LEF compared to the baseline. However, these observations were not statistically significant (**Table 3**). After adjusting for age, gender, disease duration, smoking status, obesity, and alcohol consumption factors, the observed trends of higher AST levels at the second and third visits in PsA patients treated with a combination of MTX and LEF compared to the baseline remained insignificant.

**Table 3.** The univariate and multivariate generalised estimating equations (GEE) analyses of the association between ALT/AST levels and methotrexate and leflunomide treatment.

	**B**	**SE**	**95% CI**	**p-value**
**Univariate analysis**				
**AST**				
Visit 1	0^a^			
Visit 2	1.92	1.71	−1.42, 5.27	0.260
Visit 3	0.67	1.98	−3.21, 4.55	0.736
**ALT**				
Visit 1	0^a^			
Visit 2	−1.82	2.33	−6.39, 2.75	0.436
Visit 3	−2.37	2.70	−7.65, 2.91	0.379
**Multivariate analysis**				
**AST**				
Visit 1	0^a^			
Visit 2	1.95	3.07	−4.06, 7.97	0.525
Visit 3	−4.88	4.58	−13.86, 4.11	0.287
**ALT**				
Visit 1	0^a^			
Visit 2	−3.74	3.05	−9.72, 2.23	0.220
Visit 3	−4.27	3.36	−10.86, 2.32	0.204

a Set to zero because this parameter is redundant. The multivariate model analysis controlled for age, gender, disease duration, smoking status, obesity, and alcohol consumption.

AST: aspartate aminotransferase; ALT: alanine aminotransferase.

## DISCUSSION

In this study, we observed that liver abnormalities were common among PsA patients, with approximately 40% of patients developing such abnormalities after their first evaluation at our clinic. Our findings are comparable to a previous study conducted by Rattapol et al., which reported a prevalence of liver abnormalities of 32% in PsA patients.^8^ The similar prevalence suggests consistency and supports the significance of liver abnormalities in this patient population.

We observed that PsA patients who were treated with combination of MTX with LEF had higher liver enzyme levels in the two subsequent visits compare to baseline levels. However, these observations did not reach statistical significance. Kremer JM et al. showed the combination use of MTX and LEF in rheumatoid arthritis patients resulted in a 4-fold increase in liver transaminase elevation compared to MTX alone.^9^ Liver-related adverse events are a known risk associated with MTX and LEF, ranging from minor transaminase increases to serious conditions like fibrosis and hepatic necrosis. ^10,11^ Liver enzyme elevations were frequently transient in nature, as any observed elevations typically prompted adjustments to the patient’s therapy or dose reduction.

Visser et al. have reported that liver enzyme abnormalities are common in patients using MTX for psoriasis or other conditions, with an incidence rate of approximately 40/100 patient-years in the first two years of use and a cumulative incidence of 49% after three years.^12^ This might explained why PsA patients with longer duration of disease are more likely to have been on drug treatments for a longer period, and hence, are at higher risk of developing liver abnormalities which in line with our research findings.

Several randomised clinical trials have reported an association between the use of tumour necrosis factor inhibitors (TNFi) and drug-induced liver injury.^13–16^ Additionally, increased production of Interleukin 17A (IL-17A) has been observed in various liver diseases.^17,18^ Therefore, targeting IL-17 in patients with psoriatic arthritis (PsA) may prevent hepatic complications such as NAFLD.^19^ In our analysis, among those PsA patients that were on biologics almost 40% were on IL-17A inhibitors. This may explain why biologics usage in our study which include TNFi, IL-17A inhibitor and interleukin 23p19 inhibitors did not show any association with liver abnormalities in PsA patients.

We found that PsA patients with diabetes mellitus (DM) were more likely to have liver abnormalities than those non-diabetic patients. Previous published study had demonstrated the significant association diabetic patients with raise liver enzymes when compare to healthy person.^20,21^ Disturbances in liver function tests are commonly observed in diabetic patients, as increased liver enzyme activity has been linked to insulin resistance and type 2 DM.^22^ DM is one of the most common comorbidities for PsA patients.^23^ Although the link between PsA and DM is not yet fully understood, TNF-α and adipokines such as adiponectin are thought to be among the principal mediators.^24^

NAFLD is a common condition affecting general population and lead to liver enzymes abnormalities.^25^ Although NAFLD is a prevalent liver disease with risk factors common to PsA, there is limited data available regarding its prevalence. Sonographic evidence of hepatic steatosis and elevated liver enzymes are commonly used as non-invasive surrogate markers for the diagnosis of NAFLD but liver biopsy remained the gold standard diagnostic tool.^26–28^ PsA patients, particularly those with severe disease exhibit elevated levels of cytokines such as tumour necrosis factor alpha (TNF-a) and interleukin-6 (IL-6) which have been implicated in the development and progression of NAFLD. ^29,30,31^ NAFLD can progress to non-alcoholic steatohepatitis (NASH), which increases the risk of developing liver fibrosis, cirrhosis, and hepatocellular carcinoma.^32^ It is prudent to conduct routine evaluations for NAFLD in PsA patients particularly those with metabolic syndrome and unexplained elevations in liver enzymes.

Our study has limitations. It is possible that some patients without elevated liver function tests may have had undiagnosed NAFLD. A more comprehensive evaluation of PsA patients for NAFLD, especially those with comorbidities, would be necessary to identify these patients and use hepatotoxic agents in treatment with caution. Further research is needed to investigate the prevalence and risk factors of NAFLD in PsA patients and to develop appropriate screening and monitoring strategies.

Our research revealed that liver abnormalities in PsA patients was linked to disease duration, diabetes mellitus, and NAFLD. Close monitoring of liver function tests is crucial in these high-risk populations as early detection and intervention are paramount.

## CONFLICT OF INTEREST

None.

## FUNDING

None.
